# Poly[tris­(μ-benzene-1,4-dicarboxylato)bis­(dipyrido[3,2-*a*:2′,3′-*c*]phenazine)trimanganese(II)]

**DOI:** 10.1107/S1600536808019752

**Published:** 2008-07-05

**Authors:** Wen-Zhi Zhang, Qun Xu

**Affiliations:** a College of Chemistry and Chemical Engineering, Qiqihar University, Qiqihar 161006, Heilongjiang Province, People’s Republic of China

## Abstract

In the title compound, [Mn_3_(C_8_H_4_O_4_)_3_(C_14_H_8_N_4_)_2_]_*n*_, one Mn atom is located on an inversion centre and is six-coordinated by four carboxyl­ate O atoms from different benzene-1,4-dicarboxyl­ate (1,4-bdc) ligands and two phenanthrene N atoms from a dipyrido[3,2-*a*:2′,3′-*c*]phenazine ligand. The other Mn atom is also six-coordinate, binding to six carboxyl­ate O atoms from different 1,4-bdc ligands. The dicarboxyl­ate groups chelate and bridge the two Mn atoms and a symmetry-related Mn atom to form a trimanganese unit. Bridging of the trinuclear Mn^II^ clusters leads to a two-dimensional structure.

## Related literature

For related structures, see: Chen & Liu (2002 ▶).
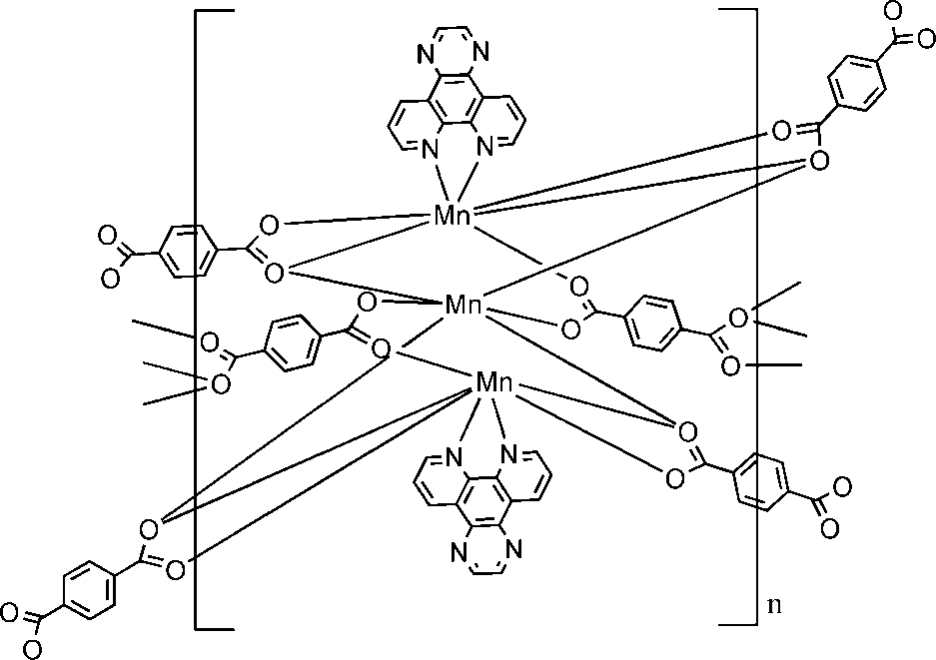


## Experimental

### 

#### Crystal data

[Mn_3_(C_8_H_4_O_4_)_3_(C_14_H_8_N_4_)_2_]
*M* *_r_* = 1121.64
Monoclinic, 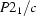

*a* = 13.323 (3) Å
*b* = 10.949 (2) Å
*c* = 16.315 (3) Å
β = 90.04 (3)°
*V* = 2379.9 (8) Å^3^
*Z* = 2
Mo *K*α radiation
μ = 0.86 mm^−1^
*T* = 293 (2) K
0.28 × 0.21 × 0.19 mm

#### Data collection

Rigaku R-AXIS RAPID diffractometer
Absorption correction: multi-scan (*ABSCOR*; Higashi, 1995 ▶) *T*_min_ = 0.783, *T*_max_ = 0.847
21939 measured reflections
5424 independent reflections
3840 reflections with *I* > 2σ(*I*)
*R*_int_ = 0.073

#### Refinement

*R*[*F* ^2^ > 2σ(*F* ^2^)] = 0.047
*wR*(*F* ^2^) = 0.098
*S* = 1.05
5424 reflections
340 parameters
H-atom parameters constrained
Δρ_max_ = 0.30 e Å^−3^
Δρ_min_ = −0.39 e Å^−3^

### 

Data collection: *PROCESS-AUTO* (Rigaku, 1998 ▶); cell refinement: *PROCESS-AUTO*; data reduction: *PROCESS-AUTO*; program(s) used to solve structure: *SHELXS97* (Sheldrick, 2008 ▶); program(s) used to refine structure: *SHELXL97* (Sheldrick, 2008 ▶); molecular graphics: *SHELXTL-Plus* (Sheldrick, 2008 ▶); software used to prepare material for publication: *SHELXL97*.

## Supplementary Material

Crystal structure: contains datablocks global, I. DOI: 10.1107/S1600536808019752/dn2359sup1.cif

Structure factors: contains datablocks I. DOI: 10.1107/S1600536808019752/dn2359Isup2.hkl

Additional supplementary materials:  crystallographic information; 3D view; checkCIF report

## Figures and Tables

**Fig. 1.**
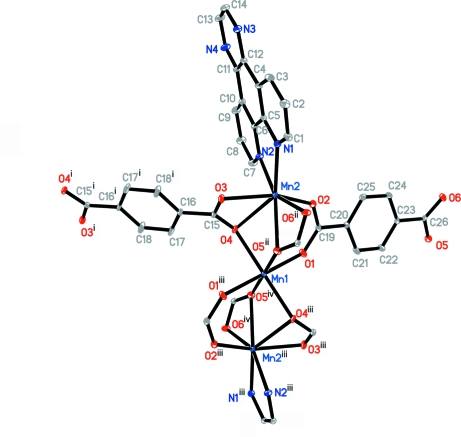
The structure of (I), showing the atomic numbering scheme. Displacement ellipsoids are drawn at the 20% probability level. H atoms and also some C atoms have been omitted for the sake of clarity. Symmetry code: (i) 1-x, 2-y, 1-z; (ii) x, 0.5-y, z-0.5; (iii) 1-x, 1-y, 1-z; (iv) 1-x, 0.5+y, 1.5-z.

## supplementary crystallographic information

### Comment

Benzene-1,4-dicarboxylic acid (1,4-H_2_bdc), as a multidentate ligand, has been extensively studied in the chemistry of coordination polymers (Chen & Liu, 2002). Here, we report a new Mn^II^ coordination polymer with 1,4-bdc ligand, namely [Mn_3_(1,4-bdc)_3_(*L*)_2_] (I), where *L* = dipyrido[3,2-a:2',3'-c]-phenazine.

In (I) the Mn1 atom is located on an inversion center and six-coordinated by four carboxylate O atoms from different 1,4-bdc ligands and two phenanthrene N atoms from *L* ligand. The Mn2 atom is also six-coordinate binding to six carboxylate O atoms from different 1,4-bdc ligands (Fig. 1). The dicarboxylato groups chelate and bridge the Mn1, Mn2 and Mn2^iii^ atoms to form a trimanganese unit (Fig. 1). The asymmetric unit based on trinuclear Mn^II^ clusters leads to a 2D structure. The L ligands are attached on both sides of the layers.

### Experimental

A mixture of Mn(NO_3_)_2_.2H_2_O (1 mmol), 1,4-H_2_bdc (1 mmol) and *L* (1 mmol) was dissolved in 12 ml distilled water, followed by addition of triethylamine until the pH value of the system was approximately 5.0. The resulting solution was sealed in a 23 ml Teflon-lined stainless steel autoclave and heated at 190 °C for 10 days under autogenous pressure. The reaction vessel was then slowly cooled to room temperature. Pale-yellow block-like crystals of (I) suitable for single-crystal X-ray diffraction analysis were obtained from the resulting solution.

### Refinement

C-bound H atoms were positioned geometrically (C—H = 0.93–0.96 Å) and refined as riding, with *U*_iso_(H) = 1.2*U*_eq_(carrier).

### Crystal data

**Table:**

[Mn_3_(C_8_H_4_O_4_)_3_(C_14_H_8_N_4_)_2_]	*F*_000_ = 1134
*M**_r_* = 1121.64	*D*_x_ = 1.565 Mg m^−^^3^
Monoclinic, *P*2_1_/*c*	Mo *K*α radiation λ = 0.71073 Å
Hall symbol: -P 2ybc	Cell parameters from 14328 reflections
*a* = 13.323 (3) Å	θ = 3.0–27.5º
*b* = 10.949 (2) Å	µ = 0.86 mm^−^^1^
*c* = 16.315 (3) Å	*T* = 293 (2) K
β = 90.04 (3)º	Block, pale yellow
*V* = 2379.9 (8) Å^3^	0.28 × 0.21 × 0.19 mm
*Z* = 2	

### Data collection

**Table:**

Rigaku R-AXIS RAPID diffractometer	5424 independent reflections
Radiation source: rotating anode	3840 reflections with *I* > 2σ(*I*)
Monochromator: graphite	*R*_int_ = 0.073
Detector resolution: 10.0 pixels mm^-1^	θ_max_ = 27.5º
*T* = 293(2) K	θ_min_ = 3.1º
ω scans	*h* = −17→17
Absorption correction: multi-scan(ABSCOR; Higashi, 1995)	*k* = −14→13
*T*_min_ = 0.783, *T*_max_ = 0.847	*l* = −21→20
21939 measured reflections	

### Refinement

**Table:**

Refinement on *F*^2^	Secondary atom site location: difference Fourier map
Least-squares matrix: full	Hydrogen site location: inferred from neighbouring sites
*R*[*F*^2^ > 2σ(*F*^2^)] = 0.047	H-atom parameters constrained
*wR*(*F*^2^) = 0.098	*w* = 1/[σ^2^(*F*_o_^2^) + (0.0202*P*)^2^ + 2.6391*P*] where *P* = (*F*_o_^2^ + 2*F*_c_^2^)/3
*S* = 1.05	(Δ/σ)_max_ = 0.001
5424 reflections	Δρ_max_ = 0.30 e Å^−^^3^
340 parameters	Δρ_min_ = −0.39 e Å^−^^3^
Primary atom site location: structure-invariant direct methods	Extinction correction: none

### Special details

**Table:**

Geometry. All esds (except the esd in the dihedral angle between two l.s. planes) are estimated using the full covariance matrix. The cell esds are taken into account individually in the estimation of esds in distances, angles and torsion angles; correlations between esds in cell parameters are only used when they are defined by crystal symmetry. An approximate (isotropic) treatment of cell esds is used for estimating esds involving l.s. planes.
Refinement. Refinement of F^2^ against ALL reflections. The weighted R-factor wR and goodness of fit S are based on F^2^, conventional R-factors R are based on F, with F set to zero for negative F^2^. The threshold expression of F^2^ > 2sigma(F^2^) is used only for calculating R-factors(gt) etc. and is not relevant to the choice of reflections for refinement. R-factors based on F^2^ are statistically about twice as large as those based on F, and R- factors based on ALL data will be even larger.

### Fractional atomic coordinates and isotropic or equivalent isotropic displacement parameters (Å^2^)

**Table:**

	*x*	*y*	*z*	*U*_iso_*/*U*_eq_	
C1	0.0322 (2)	0.6320 (3)	0.4463 (2)	0.0404 (8)	
H1	0.0486	0.5660	0.4130	0.048*	
C2	−0.0586 (3)	0.6896 (3)	0.4341 (2)	0.0482 (9)	
H2	−0.1025	0.6616	0.3939	0.058*	
C3	−0.0840 (3)	0.7882 (3)	0.4815 (2)	0.0459 (9)	
H3	−0.1446	0.8286	0.4733	0.055*	
C4	−0.0176 (2)	0.8268 (3)	0.54190 (19)	0.0328 (7)	
C5	0.0715 (2)	0.7628 (3)	0.55150 (18)	0.0287 (7)	
C6	0.1417 (2)	0.7963 (3)	0.61634 (18)	0.0289 (7)	
C7	0.2902 (3)	0.7568 (3)	0.6832 (2)	0.0433 (8)	
H7	0.3489	0.7113	0.6878	0.052*	
C8	0.2726 (3)	0.8488 (3)	0.7401 (2)	0.0494 (9)	
H8	0.3187	0.8648	0.7815	0.059*	
C9	0.1856 (3)	0.9157 (3)	0.7339 (2)	0.0465 (9)	
H9	0.1719	0.9771	0.7717	0.056*	
C10	0.1185 (2)	0.8913 (3)	0.67094 (19)	0.0338 (7)	
C11	−0.0389 (2)	0.9296 (3)	0.5959 (2)	0.0361 (7)	
C12	0.0252 (2)	0.9581 (3)	0.6599 (2)	0.0374 (8)	
C13	−0.0781 (3)	1.1090 (4)	0.6994 (3)	0.0612 (11)	
H13	−0.0953	1.1723	0.7348	0.073*	
C14	−0.1422 (3)	1.0841 (3)	0.6341 (3)	0.0570 (11)	
H14	−0.1990	1.1323	0.6267	0.068*	
C15	0.4005 (2)	0.7660 (3)	0.49542 (19)	0.0324 (7)	
C16	0.4529 (2)	0.8864 (2)	0.4964 (2)	0.0335 (7)	
C17	0.5458 (3)	0.8984 (3)	0.5301 (3)	0.0619 (12)	
H17	0.5784	0.8299	0.5508	0.074*	
C18	0.5926 (3)	1.0109 (3)	0.5339 (3)	0.0664 (13)	
H18	0.6559	1.0170	0.5575	0.080*	
C19	0.3513 (2)	0.4088 (3)	0.63506 (18)	0.0311 (7)	
C20	0.3366 (2)	0.3233 (3)	0.70612 (18)	0.0297 (7)	
C21	0.4127 (2)	0.2443 (3)	0.7294 (2)	0.0442 (9)	
H21	0.4735	0.2450	0.7015	0.053*	
C22	0.3987 (2)	0.1647 (3)	0.7938 (2)	0.0388 (8)	
H22	0.4505	0.1130	0.8099	0.047*	
C23	0.3081 (2)	0.1615 (2)	0.83462 (17)	0.0259 (6)	
C24	0.2313 (2)	0.2385 (3)	0.8108 (2)	0.0366 (8)	
H24	0.1696	0.2349	0.8372	0.044*	
C25	0.2460 (2)	0.3212 (3)	0.74757 (19)	0.0368 (8)	
H25	0.1951	0.3751	0.7330	0.044*	
C26	0.2900 (2)	0.0716 (2)	0.90190 (17)	0.0253 (6)	
N1	0.09707 (18)	0.6666 (2)	0.50352 (15)	0.0314 (6)	
N2	0.22697 (19)	0.7308 (2)	0.62252 (16)	0.0331 (6)	
N3	−0.1245 (2)	0.9939 (3)	0.58228 (19)	0.0483 (8)	
N4	0.0048 (2)	1.0488 (3)	0.7141 (2)	0.0531 (8)	
O1	0.43683 (17)	0.4150 (2)	0.60481 (14)	0.0475 (6)	
O2	0.27522 (16)	0.46763 (19)	0.61136 (13)	0.0367 (5)	
O3	0.31820 (17)	0.75629 (19)	0.45763 (15)	0.0401 (6)	
O4	0.43671 (16)	0.67877 (17)	0.53665 (13)	0.0333 (5)	
O5	0.36377 (16)	0.01648 (19)	0.93248 (13)	0.0370 (5)	
O6	0.20305 (16)	0.0478 (2)	0.92461 (13)	0.0395 (6)	
Mn1	0.5000	0.5000	0.5000	0.02269 (14)	
Mn2	0.25609 (3)	0.59443 (4)	0.51712 (3)	0.02279 (11)	

### Atomic displacement parameters (Å^2^)

**Table:**

	*U*^11^	*U*^22^	*U*^33^	*U*^12^	*U*^13^	*U*^23^
C1	0.0354 (19)	0.047 (2)	0.039 (2)	−0.0036 (15)	−0.0036 (15)	−0.0090 (15)
C2	0.036 (2)	0.064 (2)	0.045 (2)	−0.0035 (18)	−0.0131 (16)	−0.0072 (18)
C3	0.0327 (19)	0.057 (2)	0.048 (2)	0.0051 (17)	−0.0031 (16)	0.0013 (17)
C4	0.0269 (16)	0.0366 (17)	0.0350 (18)	0.0020 (14)	0.0016 (13)	0.0037 (13)
C5	0.0292 (16)	0.0252 (15)	0.0317 (17)	0.0002 (12)	0.0033 (13)	0.0015 (12)
C6	0.0302 (16)	0.0241 (15)	0.0323 (17)	0.0035 (12)	0.0018 (13)	0.0002 (12)
C7	0.039 (2)	0.050 (2)	0.041 (2)	0.0085 (16)	−0.0090 (15)	−0.0107 (16)
C8	0.047 (2)	0.056 (2)	0.045 (2)	0.0042 (18)	−0.0160 (17)	−0.0207 (17)
C9	0.051 (2)	0.044 (2)	0.045 (2)	0.0041 (17)	−0.0037 (16)	−0.0180 (16)
C10	0.0363 (18)	0.0304 (17)	0.0348 (18)	0.0003 (14)	0.0012 (13)	−0.0014 (13)
C11	0.0318 (17)	0.0314 (18)	0.045 (2)	0.0060 (14)	0.0048 (14)	0.0062 (14)
C12	0.0397 (19)	0.0298 (17)	0.043 (2)	0.0064 (14)	0.0072 (15)	−0.0030 (14)
C13	0.061 (3)	0.046 (2)	0.077 (3)	0.020 (2)	0.006 (2)	−0.014 (2)
C14	0.049 (2)	0.042 (2)	0.079 (3)	0.0222 (19)	0.009 (2)	0.003 (2)
C15	0.0368 (18)	0.0235 (16)	0.0368 (18)	−0.0017 (13)	0.0047 (14)	−0.0001 (13)
C16	0.0309 (17)	0.0184 (15)	0.051 (2)	−0.0031 (13)	−0.0035 (14)	0.0056 (13)
C17	0.051 (2)	0.0238 (18)	0.111 (4)	−0.0033 (17)	−0.034 (2)	0.021 (2)
C18	0.049 (2)	0.0296 (19)	0.121 (4)	−0.0141 (18)	−0.042 (2)	0.020 (2)
C19	0.0385 (18)	0.0289 (16)	0.0258 (16)	−0.0024 (14)	0.0006 (13)	0.0048 (12)
C20	0.0344 (17)	0.0291 (16)	0.0256 (16)	−0.0025 (13)	−0.0002 (12)	0.0079 (12)
C21	0.0293 (18)	0.056 (2)	0.047 (2)	0.0019 (16)	0.0076 (15)	0.0238 (17)
C22	0.0276 (17)	0.048 (2)	0.041 (2)	0.0079 (15)	0.0021 (14)	0.0201 (15)
C23	0.0305 (16)	0.0253 (15)	0.0220 (15)	−0.0025 (12)	−0.0021 (12)	0.0036 (11)
C24	0.0335 (18)	0.0392 (18)	0.0371 (19)	0.0059 (14)	0.0103 (14)	0.0148 (14)
C25	0.0405 (19)	0.0358 (18)	0.0342 (18)	0.0115 (15)	0.0058 (14)	0.0110 (14)
C26	0.0330 (16)	0.0207 (14)	0.0223 (15)	−0.0003 (12)	−0.0008 (12)	0.0001 (11)
N1	0.0318 (14)	0.0299 (14)	0.0324 (15)	−0.0004 (11)	−0.0022 (11)	−0.0025 (11)
N2	0.0339 (15)	0.0334 (14)	0.0320 (15)	0.0071 (11)	−0.0013 (11)	−0.0065 (11)
N3	0.0420 (17)	0.0420 (17)	0.061 (2)	0.0127 (14)	0.0055 (14)	0.0061 (15)
N4	0.054 (2)	0.0421 (17)	0.064 (2)	0.0163 (15)	0.0024 (16)	−0.0168 (15)
O1	0.0393 (13)	0.0572 (16)	0.0462 (15)	0.0007 (12)	0.0114 (11)	0.0291 (12)
O2	0.0418 (13)	0.0353 (12)	0.0329 (13)	0.0052 (10)	0.0036 (10)	0.0149 (9)
O3	0.0385 (13)	0.0260 (11)	0.0560 (15)	−0.0088 (10)	−0.0080 (11)	0.0055 (10)
O4	0.0479 (13)	0.0159 (10)	0.0361 (12)	−0.0008 (9)	0.0007 (10)	0.0003 (8)
O5	0.0363 (12)	0.0400 (13)	0.0349 (13)	0.0057 (10)	−0.0038 (10)	0.0153 (10)
O6	0.0340 (13)	0.0456 (13)	0.0391 (13)	−0.0035 (10)	0.0021 (10)	0.0213 (10)
Mn1	0.0237 (3)	0.0192 (3)	0.0252 (3)	−0.0012 (2)	0.0033 (2)	0.0028 (2)
Mn2	0.0281 (2)	0.0183 (2)	0.0220 (2)	0.00027 (18)	0.00073 (16)	−0.00085 (17)

### Geometric parameters (Å, °)

**Table:**

C1—N1	1.328 (4)	C17—H17	0.9300
C1—C2	1.379 (5)	C18—C16^i^	1.369 (4)
C1—H1	0.9300	C18—H18	0.9300
C2—C3	1.370 (5)	C19—O1	1.244 (4)
C2—H2	0.9300	C19—O2	1.262 (4)
C3—C4	1.390 (4)	C19—C20	1.503 (4)
C3—H3	0.9300	C20—C25	1.384 (4)
C4—C5	1.387 (4)	C20—C21	1.386 (4)
C4—C11	1.457 (4)	C21—C22	1.378 (4)
C5—N1	1.356 (4)	C21—H21	0.9300
C5—C6	1.458 (4)	C22—C23	1.379 (4)
C6—N2	1.347 (4)	C22—H22	0.9300
C6—C10	1.404 (4)	C23—C24	1.382 (4)
C7—N2	1.331 (4)	C23—C26	1.494 (4)
C7—C8	1.390 (5)	C24—C25	1.386 (4)
C7—H7	0.9300	C24—H24	0.9300
C8—C9	1.374 (5)	C25—H25	0.9300
C8—H8	0.9300	C26—O6	1.245 (3)
C9—C10	1.388 (4)	C26—O5	1.256 (3)
C9—H9	0.9300	N1—Mn2	2.272 (3)
C10—C12	1.454 (4)	N2—Mn2	2.311 (2)
C11—N3	1.358 (4)	O1—Mn1	2.121 (2)
C11—C12	1.385 (5)	O2—Mn2	2.087 (2)
C12—N4	1.357 (4)	O3—Mn2	2.184 (2)
C13—N4	1.309 (5)	O4—Mn1	2.214 (2)
C13—C14	1.392 (6)	O5—Mn1^ii^	2.130 (2)
C13—H13	0.9300	O5—Mn2^iii^	2.333 (2)
C14—N3	1.322 (5)	O6—Mn2^iii^	2.281 (2)
C14—H14	0.9300	Mn1—O1^iv^	2.121 (2)
C15—O3	1.262 (4)	Mn1—O5^v^	2.130 (2)
C15—O4	1.264 (3)	Mn1—O5^vi^	2.130 (2)
C15—C16	1.492 (4)	Mn1—O4^iv^	2.214 (2)
C16—C17	1.361 (4)	Mn2—O6^v^	2.281 (2)
C16—C18^i^	1.369 (4)	Mn2—O5^v^	2.333 (2)
C17—C18	1.382 (5)		
			
N1—C1—C2	122.8 (3)	C20—C21—H21	119.9
N1—C1—H1	118.6	C21—C22—C23	120.2 (3)
C2—C1—H1	118.6	C21—C22—H22	119.9
C3—C2—C1	119.8 (3)	C23—C22—H22	119.9
C3—C2—H2	120.1	C22—C23—C24	119.8 (3)
C1—C2—H2	120.1	C22—C23—C26	120.8 (3)
C2—C3—C4	118.8 (3)	C24—C23—C26	119.3 (3)
C2—C3—H3	120.6	C23—C24—C25	120.2 (3)
C4—C3—H3	120.6	C23—C24—H24	119.9
C5—C4—C3	118.1 (3)	C25—C24—H24	119.9
C5—C4—C11	119.2 (3)	C20—C25—C24	119.9 (3)
C3—C4—C11	122.6 (3)	C20—C25—H25	120.1
N1—C5—C4	122.9 (3)	C24—C25—H25	120.1
N1—C5—C6	116.9 (3)	O6—C26—O5	120.6 (3)
C4—C5—C6	120.2 (3)	O6—C26—C23	120.5 (3)
N2—C6—C10	122.2 (3)	O5—C26—C23	118.8 (3)
N2—C6—C5	117.5 (3)	C1—N1—C5	117.6 (3)
C10—C6—C5	120.3 (3)	C1—N1—Mn2	125.2 (2)
N2—C7—C8	123.0 (3)	C5—N1—Mn2	116.66 (19)
N2—C7—H7	118.5	C7—N2—C6	118.4 (3)
C8—C7—H7	118.5	C7—N2—Mn2	125.8 (2)
C9—C8—C7	118.7 (3)	C6—N2—Mn2	115.48 (19)
C9—C8—H8	120.6	C14—N3—C11	115.7 (3)
C7—C8—H8	120.6	C13—N4—C12	114.7 (3)
C8—C9—C10	119.6 (3)	C19—O1—Mn1	135.1 (2)
C8—C9—H9	120.2	C19—O2—Mn2	131.5 (2)
C10—C9—H9	120.2	C15—O3—Mn2	100.40 (19)
C9—C10—C6	118.1 (3)	C15—O4—Mn1	132.0 (2)
C9—C10—C12	123.0 (3)	C26—O5—Mn1^ii^	155.8 (2)
C6—C10—C12	118.9 (3)	C26—O5—Mn2^iii^	90.23 (17)
N3—C11—C12	121.5 (3)	Mn1^ii^—O5—Mn2^iii^	100.02 (8)
N3—C11—C4	117.7 (3)	C26—O6—Mn2^iii^	92.95 (17)
C12—C11—C4	120.7 (3)	O1^iv^—Mn1—O1	180.0
N4—C12—C11	122.2 (3)	O1^iv^—Mn1—O5^v^	87.63 (9)
N4—C12—C10	117.3 (3)	O1—Mn1—O5^v^	92.37 (9)
C11—C12—C10	120.4 (3)	O1^iv^—Mn1—O5^vi^	92.37 (9)
N4—C13—C14	124.0 (4)	O1—Mn1—O5^vi^	87.63 (9)
N4—C13—H13	118.0	O5^v^—Mn1—O5^vi^	180.00 (10)
C14—C13—H13	118.0	O1^iv^—Mn1—O4^iv^	91.07 (9)
N3—C14—C13	121.8 (3)	O1—Mn1—O4^iv^	88.93 (9)
N3—C14—H14	119.1	O5^v^—Mn1—O4^iv^	96.31 (8)
C13—C14—H14	119.1	O5^vi^—Mn1—O4^iv^	83.69 (8)
O3—C15—O4	121.8 (3)	O1^iv^—Mn1—O4	88.93 (9)
O3—C15—C16	119.1 (3)	O1—Mn1—O4	91.07 (9)
O4—C15—C16	119.0 (3)	O5^v^—Mn1—O4	83.69 (8)
C17—C16—C18^i^	117.9 (3)	O5^vi^—Mn1—O4	96.31 (8)
C17—C16—C15	121.0 (3)	O4^iv^—Mn1—O4	180.00 (10)
C18^i^—C16—C15	121.0 (3)	O2—Mn2—O3	145.20 (9)
C16—C17—C18	121.0 (3)	O2—Mn2—N1	114.63 (9)
C16—C17—H17	119.5	O3—Mn2—N1	91.60 (9)
C18—C17—H17	119.5	O2—Mn2—O6^v^	94.06 (9)
C16^i^—C18—C17	121.1 (3)	O3—Mn2—O6^v^	112.18 (9)
C16^i^—C18—H18	119.5	N1—Mn2—O6^v^	83.37 (9)
C17—C18—H18	119.5	O2—Mn2—N2	84.38 (9)
O1—C19—O2	125.9 (3)	O3—Mn2—N2	82.54 (9)
O1—C19—C20	117.4 (3)	N1—Mn2—N2	71.99 (9)
O2—C19—C20	116.7 (3)	O6^v^—Mn2—N2	151.86 (9)
C25—C20—C21	119.6 (3)	O2—Mn2—O5^v^	90.86 (9)
C25—C20—C19	120.1 (3)	O3—Mn2—O5^v^	85.76 (9)
C21—C20—C19	120.3 (3)	N1—Mn2—O5^v^	134.20 (8)
C22—C21—C20	120.3 (3)	O6^v^—Mn2—O5^v^	56.17 (7)
C22—C21—H21	119.9	N2—Mn2—O5^v^	151.71 (8)

Symmetry codes: (i) −*x*+1, −*y*+2, −*z*+1; (ii) −*x*+1, *y*−1/2, −*z*+3/2; (iii) *x*, −*y*+1/2, *z*+1/2; (iv) −*x*+1, −*y*+1, −*z*+1; (v) *x*, −*y*+1/2, *z*−1/2; (vi) −*x*+1, *y*+1/2, −*z*+3/2.
